# Two barriers for sodium in vascular endothelium?

**DOI:** 10.3109/07853890.2011.653397

**Published:** 2012-04-03

**Authors:** Hans Oberleithner

**Affiliations:** Institute of Physiology II, University of Münster, Germany

**Keywords:** Aldosterone, atomic force microscopy, endothelial dysfunction, endothelial glycocalyx, epithelial sodium channel, hypertension, mechanical stiffness, salt intake, spironolactone, stiff endothelial cell syndrome

## Abstract

Vascular endothelium plays a key role in blood pressure regulation. Recently, it has been shown that a 5% increase of plasma sodium concentration (sodium excess) stiffens endothelial cells by about 25%, leading to cellular dysfunction. Surface measurements demonstrated that the endothelial glycocalyx (eGC), an anionic biopolymer, deteriorates when sodium is elevated. In view of these results, a two-barrier model for sodium exiting the circulation across the endothelium is suggested. The first sodium barrier is the eGC which selectively buffers sodium ions with its negatively charged prote-oglycans.The second sodium barrier is the endothelial plasma membrane which contains sodium channels. Sodium excess, in the presence of aldosterone, leads to eGC break-down and, in parallel, to an up-regulation of plasma membrane sodium channels. The following hypothesis is postulated: Sodium excess increases vascular sodium permeability. Under such con-ditions (e.g. high-sodium diet), day-by-day ingested sodium, instead of being readily buffered by the eGC and then rapidly excreted by the kidneys, is distributed in the whole body before being finally excreted. Gradually, the sodium overload damages the organism.

## Endothelial cell mechanics

In life, mechanical force is applied to most tissues, particularly to vascular endothelium. Hemodynamic forces, generated by the heart's contraction, give rise to shear stress at the endothelial surface. It is inevitable, therefore, that the luminal cell surface undergoes pulsatile reversible deformation. It is this mechanical stimulus which triggers the activity of endothelial nitric oxide synthase (eNOS) and the release of nitric oxide (1,2). NO diffuses to the adjacent vascular smooth muscle which relaxes, leading to vasodilation. In mechanically stiff, less deformable cells, shear stress is expected to be only poorly effective in releasing NO. Therefore, endothelial cell stiffness could prove to be a key function in the control of tissue perfusion and blood pressure (3–5).

The ‘tool of choice’ for quantifying stiffness is an atomic force microscope (AFM). In principle, the AFM is used as a mechanical tool (6). The tip of the AFM (sometimes shaped as a small sphere) is pressed against the cell so that the membrane is indented. This distorts the cantilever on the AFM which serves as a soft spring. The cantilever deflection, measured by a laser beam, is reflected from the gold-coated cantilever surface, which permits force/distance curves of single cells to be measured. The slope of such curves is directly related to the force necessary to indent the cell for a given distance (7–9).

Caution should be exercised, however, when interpreting force/distance data of the glycocalyx. The AFM tip physically interferes with the sample surface and thus with the negatively charged glycocalyx. As a result, the stiffness/height measurements probably include an ‘electrical component’ of unknown magnitude. In other words, repulsive/attractive forces between tip and sample may play a role in such measurements. This could explain some of the differences in glycocalyx thickness measurements performed in different preparations (e.g. *in vitro*, *ex vivo*, *in vivo*), in different conditions (e.g. static condition versus shear stress), and with different techniques (optical versus atomic force microscopy).

**Key messages**

- The endothelial glycocalyx, a negatively charged biopolymer on the cell surface, is probably a buffer barrier for sodium.
- *In vitro*, high ambient sodium leads to the deterioration of the endothelial glycocalyx and to the enhanced insertion of sodium channels in the endothelial plasma mem-brane.
- It is postulated that the endothelial sodium permeability plays a role in the rate of renal sodium excretion.

## Sodium acts on endothelium

**Figure 1:**
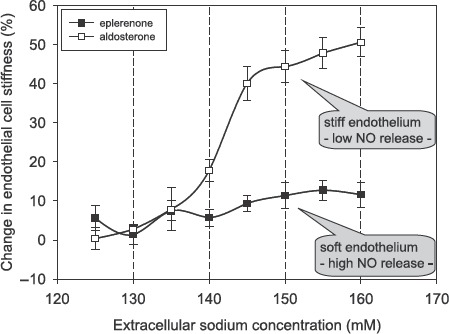
Changes in endothelial cell stiffness in response to increasing sodium concentration measured in buffered electrolyte solution (constant osmolality). Endothelial cells were cultured 3 days prior to the experiments in either aldosterone (0.45 nM) containing or eplerenone (2 μM) containing media. Stiffness measurements were started with 120 mM sodium in the solution (reference solution). Exposure time to one individual sodium concentration was about 3 minutes. Stiffness measurements obtained in cells bathed with 120 mM sodium served as the respective reference values. Mean values of ten independent measurements per series of experiments are given ± SEM (modified from (22)).

Hypertension, stroke, coronary heart disease, and renal fibrosis are related to high sodium intake (10–14). Although the deleterious effects of high sodium intake are well defined, the underlying mechanisms are not clear. A high sodium intake causes fibrosis and inflammatory processes in kidney and heart (15). In addition, C-reactive protein (CRP), known as a marker for inflammation, potentiates the aldosterone-induced stiff endothelial cell syndrome (SECS), indicating that sodium is involved in this process (16,17). When the rate of dietary salt intake tempo-rarily exceeds renal excretory capacity, some of this increase is stored in the space between cells, bound to extracellular organic material (18).Titze et al. (19) were first to relate osmotically inactive sodium stores to arterial hypertension. Plasma sodium is slightly increased in hypertension when dietary sodium intake is raised (20,21). It has therefore been postulated that changes in plasma sodium may control blood pres-sure (21). *In-vitro* experiments have shown that when extracellular sodium concentration is raised by about 5% endothelial cells stiffen within minutes by up to 25% (22). Figure 1 illustrates the large impact of small changes of extracellular sodium concentration on vascular endothelial stiffness.This strong response of the endothelium to small changes in sodium concentration is dependent upon aldosterone. Inhibi-tion of the cytosolic mineralocorticoid receptors by spironolactone (or eplerenone) prevents endothelial stiffening. Taken together, small changes in plasma sodium can alter endothelial function, at least *in vitro*. A major prerequisite for this sodium-induced change in endothelial function is aldosterone, which is usu-ally present under physiological conditions (more details in (22)).

## Endothelial glycocalyx (eGC)

**Figure 2:**
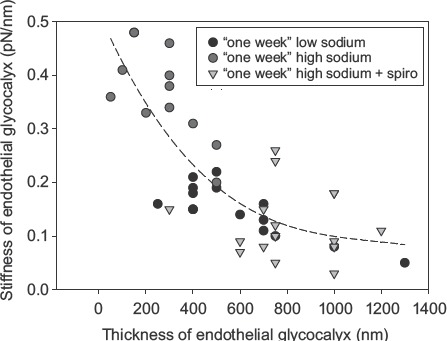
Correlation between eGC thickness and eGC stiffness. Experiments were performed in arteries *ex vivo* exposed for 1 week in culture conditions to low (135 mM) sodium, high (150 mM) sodium, and high sodium plus spironolactone (100 nM). Low-sodium treatment results in a thick and soft eGC. High-sodium treatment results in reduced eGC thickness and increased eGC stiffness. The latter effects are blocked by the aldosterone antagonist spironolactone. Aldosterone (0.1 nM) was present in all media (modified from (31)).

The eGC participates in the regulation of vascular permeability (23,24), in the control of flow- and pressure- induced mechanotransduction of the endothelium (25,26), and may play a crucial role in the pathogenesis of inflammation (27,28). Chem-ically, the eGC is an anionic biopolymer with specific ion-binding properties (29). Proteoheparan sulphate macromolecules, anchored in the plasma membrane, expose negatively charged glycosamin-oglycan side chains with binding sites for inorganic cations. Probably, calcium ions control eGC con-formation in a way that sodium ions are preferen-tially adsorbed (29,30). It has been shown that sodium excess, mimicked in *ex-vivo* experiments by a 10% increase of extracellular sodium, leads to the deterioration of the eGC (31). That aldosterone is a mediator of this process is probably due to the inser-tion of sodium channels into the plasma membrane (32–34). Deterioration of the eGC allows sodium to enter readily into the cells via the sodium channels, where it disturbs cellular function.The relationship between thickness and stiffness of the eGC is illus-trated in Figure 2. A stiff eGC, as observed in sodium excess (i.e. elevation of extracellular sodium over days), is supposedly a deteriorated eGC lack-ing heparan sulphate residues and thus exhibiting a reduced sodium buffer capacity (31).

It is possible that the eGC plays a prominent role as a buffer barrier for sodium. In a previous *in-vitro* study (31) the eGC sodium store for endothelial monolayers, grown under low (135 mM) extracel-lular sodium conditions, was measured to be 16 nmol/cm^2^. When this measurement is extrapolated to man, assuming that the total endothelial surface is 350 m^2^ and the glycocalyx thickness is 300 nm (35), then about 0.7 g of sodium (about 35 mmol) is calculated for the total amount of sodium being retained by the negative charges of the eGC. This storage capacity is reduced by about two-thirds (to about 12 mmol) after sodium excess (i.e. exposure of the *ex-vivo* endothelium to 150 mM sodium for 5 days). These calculations are quite inaccurate and probably underestimate eGC sodium storage capacity because, among other things, they are based on *in-vitro* experiments, and we know that the glycocalyx *in vitro* is less well developed than *in vivo* (36,37).

Taken together, the eGC can probably serve as a rather efficient sodium buffering barrier, though it deteriorates when chronically exposed to high sodium.

## A hypothesis on sodium homeostasis

**Figure 3:**
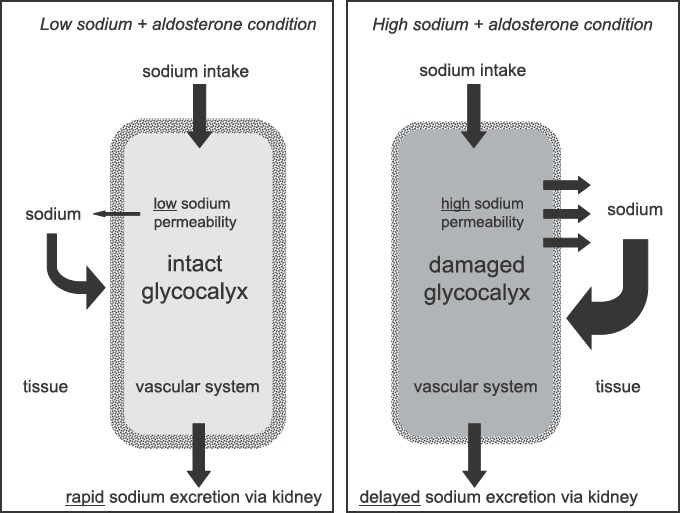
Hypothesis how endothelial sodium permeability could contribute to sodium homeostasis in the human organism. Shown are two extreme samples. On the left, the vascular system (in green) is shown at ‘low-sodium condition’ indicating an organism on ‘low-salt diet’. On the right, the vascular system (in red) is shown at ‘high-sodium condition’ indicating an organism on ‘high-salt diet’.

How fast is sodium eliminated by the kidneys, after ingestion of a salty meal, and what role does the endothelium play in this context? Figure 3 presents a hypothesis. It is assumed that an intact glycocalyx has a sodium-buffering capacity of about 35 mmol (see above), which is about the amount of sodium found in a salty snack. Two extreme examples illus-trate what may happen after ingestion of this amount of salt: The first example is an individual on a low-salt diet, i.e. with an intact glycocalyx and a low expression of sodium channels in the endothelium. When the ingested sodium arrives in the circulation it will tend to raise plasma sodium where it will be retained for a transient period. This is due to a sufficient buffering capacity of the eGC (first barrier) and the relatively low sodium permeability of the endothelial cells (second barrier). Subsequently it is eliminated by the kidneys. The second example is an individual on a high-salt diet, i.e. with a deterio-rated eGC and a high expression of sodium chan-nels in the endothelium. In contrast to the first example, in this case the same amount of ingested sodium arriving in the circulation will easily exit the vascular system due to the insufficient buffer capac-ity of the deteriorated eGC and the high sodium permeability of the endothelial cells. Thus, in these circumstances sodium is readily distributed in the large interstitial compartment of the whole body where it is transiently bound, osmotically inactive, to the negative charges of the extracellular matrix (19).Thus, the ingested sodium will escape immedi-ate renal elimination. Then, it will gradually diffuse from the interstitium back into the circulation so that its excretion by the kidneys is delayed. This potential sequence of events is supported by two observations *in vitro*, namely that vascular endothe-lium exposed to ambient high sodium exhibits 1) an increased sensitivity to the sodium channel blocker amiloride (22,38), and 2) an increased sodium per-meability after the eGC is destroyed by heparinase treatment (38,39).

The hypothesis, as described, is entirely based on *in-vitro* experiments. It will be necessary to test it in man. Furthermore, the vascular endothelium has been treated as being formed by a uniform cell type, which does not apply in man.

## Possible physiological implications

The endothelial monolayer lining the vascular sys-tem exerts a large number of different permeabilities depending on the specific function in the various organs and tissues (40,41). For example, in the kid-ney the endothelium of the glomeruli is fenestrated and thus highly permeable, whereas the endothelium of the blood–brain barrier tends to be tight. In addition, endothelial permeabilities in the different tissues are strongly regulated. The passage rate of molecules across the endothelial wall depends on molecular size, electrical charge, and lipid solubility. Electrolytes are considered to diffuse freely along their chemical gradients from blood to interstitium and vice versa. The major route for water and elec-trolytes is supposed to be the paracellular pathway between the cells. Nevertheless, endothelial cells themselves also exert specific ‘cellular’ permeabilities that are due to the lipophilicity of the plasma mem-branes and to the abundance of ion channels and transporters. In the hypothetical model described here the focus has been put on the endothelial cells, which apparently neglects the paracellular route. This approach has been used for the sake of clarity. Most likely, the transcellular route is much less per-meable for sodium than the paracellular route. Recent *in-vitro* experiments in cultured endothelial monolayers indicate that the glycocalyx may contrib-ute about 11% to the total sodium permeability of the endothelium (39). Furthermore, experimental evidence for transcellular endothelial sodium trans-port does exist because changing the ambient sodium concentration can immediately affect intracellular sodium concentration (38). There is little evidence for polarized electrolyte transport in a manner similar to epithelia (41). Assuming that endothelial cells can exert a specific sodium permeability which may depend on the glycocalyx, plasma sodium, aldoster-one, and other still unknown factors, then a variable fraction of sodium appearing in the plasma, after sodium ingestion, will transit the endothelial cell. This transcellular fraction of sodium transported may be negligibly small in some parts of the vascular system but significant in others. In addition, small changes in extracellular sodium may significantly alter endothelial cell mechanics and thus cellular function (22).

## Possible clinical implications

Sodium ‘abuse’ worldwide causes severe health problems. There is evidence that a high sodium intake weakens the protective eGC buffer barrier and increases vascular sodium permeability. This implies that sodium does not freely diffuse across the endothelium but that its diffusion is influenced by the glycocalyx and the number of endothelial sodium channels. The structural components of the eGC do not only interact with those plasma proteins which are pertinent for fluid homeosta-sis, as shown recently (28,42), but also with small electrolytes, in particular with sodium. The high sodium content, gradually accumulating in the body of individuals on high salt intake, finally damages the individual (43).

## Abbreviations

AFM: atomic force microscopy
CRP: C-reactive protein
eGC: endothelial glycocalyx
eNOS: endothelial nitric oxide synthase
NO: nitric oxide
SECS: stiff endothelial cell syndrome

## References

[1] Fleming I, Busse R (2003). Molecular mechanisms involved in the regulation of the endothelial nitric oxide synthase. Am J Physiol Regul Integr Comp Physiol.

[2] Fels J, Callies C, Kusche-Vihrog K, Oberleithner H (2010). Nitric oxide release follows endothelial nanomechanics and not vice versa. Pflugers Arch.

[3] Jonas M, So PT, Huang H (2008). Cell mechanics at multiple scales. Methods Enzymol.

[4] Kliche K, Jeggle P, Pavenstadt H, Oberleithner H (2011). Role of cellular mechanics in the function and life span of vascular endothelium. Pflugers Arch.

[5] Bussemaker E, Hillebrand U, Hausberg M, Pavenstadt H, Oberleithner H (2010). Pathogenesis of hypertension: interactions among sodium, potassium, and aldosterone. Am J Kidney Dis.

[6] Binnig G, Quate CF, Gerber C (1986). Atomic force microscope. Phys Rev Lett.

[7] Kasas S, Dietler G (2008). Probing nanomechanical properties from biomolecules to living cells. Pflugers Arch.

[8] Oberleithner H, Callies C, Kusche-Vihrog K, Schillers H, Shahin V, Riethmuller C (2009). Potassium softens vascular endothelium and increases nitric oxide release. Proc Natl Acad Sci USA.

[9] Radmacher M (1997). Measuring the elastic properties of biological samples with the AFM [see comments]. IEEE Eng Med Biol Mag.

[10] Meneton P, Jeunemaitre X, de Wardener HE, MacGregor GA (2005). Links between dietary salt intake, renal salt handling, blood pressure, and cardiovascular diseases. Physiol Rev.

[11] Adrogue HJ, Madias NE (2007). Sodium and potassium in the pathogenesis of hypertension. N Engl J Med.

[12] Funder JW (2006). Mineralocorticoid receptors and cardiovascular damage: it's not just aldosterone. Hypertension.

[13] He FJ, Marciniak M, Markandu ND, Antonios TF, MacGregor GA (2010). Effect of modest salt reduction on skin capillary rarefaction in white, black, and Asian individuals with mild hypertension. Hypertension.

[14] Ritz E (2006). Salt — friend or foe?. Nephrol Dial Transplant.

[15] Sanders PW (2009). Vascular consequences of dietary salt intake. Am J Physiol Renal Physiol.

[16] Lang F (2011). Stiff endothelial cell syndrome in vascular inflammation and mineralocorticoid excess. Hypertension.

[17] Kusche-Vihrog K, Urbanova K, Blanque A, Wilhelmi M, Schillers H, Kliche K (2011). C-reactive protein makes human endothelium stiff and tight. Hypertension.

[18] Titze J, Lang R, Ilies C, Schwind KH, Kirsch KA, Dietsch P (2003). Osmotically inactive skin Na +storage in rats. Am J Physiol Renal Physiol.

[19] Titze J, Machnik A (2010). Sodium sensing in the interstitium and relationship to hypertension. Curr Opin Nephrol Hypertens.

[20] Adams JM, Bardgett ME, Stocker SD (2009). Ventral lamina termi-nalis mediates enhanced cardiovascular responses of rostral ventrolateral medulla neurons during increased dietary salt. Hypertension.

[21] He FJ, Markandu ND, Sagnella GA, de Wardener HE, MacGregor GA (2005). Plasma sodium: ignored and underestimated. Hypertension.

[22] Oberleithner H, Riethmuller C, Schillers H, MacGregor GA, de Wardener HE, Hausberg M (2007). Plasma sodium stiffens vascular endothelium and reduces nitric oxide release. Proc Natl Acad Sci USA.

[23] Singh A, Satchell SC, Neal CR, McKenzie EA, Tooke JE, Mathieson PW (2007). Glomerular endothelial glycocalyx consti-tutes a barrier to protein permeability. J Am Soc Nephrol.

[24] VanTeeffelen JW, Brands J, Jansen C, Spaan JA, Vink H (2007). Heparin impairs glycocalyx barrier properties and attenu-ates shear dependent vasodilation in mice. Hypertension.

[25] Weinbaum S, Zhang X, Han Y, Vink H, Cowin SC (2003). Mech-anotransduction and flow across the endothelial glycocalyx. Proc Natl Acad Sci USA.

[26] Tarbell JM, Ebong EE (2008). The endothelial glycocalyx: a mechano-sensor and -transducer. Sci Signal.

[27] Nieuwdorp M, Meuwese MC, Vink H, Hoekstra JB, Kastelein JJ, Stroes ES (2005). The endothelial glycocalyx: a potential barrier between health and vascular disease. Curr Opin Lipidol.

[28] Chappell D, Westphal M, Jacob M (2009). The impact of the gly-cocalyx on microcirculatory oxygen distribution in critical illness. Curr Opin Anaesthesiol.

[29] Siegel G, Walter A, Kauschmann A, Malmsten M, Buddecke E (1996). Anionic biopolymers as blood flow sensors. Biosens Bioelectron.

[30] Bevan JA (1993). Flow regulation of vascular tone. Its sensitivity to changes in sodium and calcium. Hypertension.

[31] Oberleithner H, Peters W, Kusche-Vihrog K, Korte S, Schillers H, Kliche K (2011). Salt overload damages the glycocalyx sodium barrier of vascular endothelium. Pflugers Arch.

[32] Kusche-Vihrog K, Sobczak K, Bangel N, Wilhelmi M, Nechyporuk-Zloy V, Schwab A (2008). Aldosterone and ami-loride alter ENaC abundance in vascular endothelium. Pflugers Arch.

[33] Golestaneh N, Klein C, Valamanesh F, Suarez G, Agarwal MK, Mirshahi M (2001). Mineralocorticoid receptor-mediated signaling regulates the ion gated sodium channel in vascular endothelial cells and requires an intact cytoskeleton. Biochem Biophys Res Commun.

[34] Chen W, Valamanesh F, Mirshahi T, Soria J, Tang R, Agarwal MK (2004). Aldosterone signaling modifies capillary formation by human bone marrow endothelial cells. Vascul Pharmacol.

[35] Pries AR, Secomb TW, Gaehtgens P (2000). The endothelial surface layer. Pflugers Arch.

[36] Potter DR, Jiang J, Damiano ER (2009). The recovery time course of the endothelial cell glycocalyx in vivo and its implications in vitro. Circ Res.

[37] Chappell D, Jacob M, Paul O, Rehm M, Welsch U, Stoeckelhuber M (2009). The glycocalyx of the human umbilical vein endothelial cell: an impressive structure ex vivo but not in culture. Circ Res.

[38] Korte S, Wiesinger A, Strater AS, Peters W, Oberleithner H, Kusche-Vihrog K (2012). Firewall function of the endothelial glycocalyx in the regulation of sodium homeostasis. Pflugers Arch.

[39] Peters W, Druppel V, Kusche-Vihrog K, Schubert C, Oberleithner H (2012). Nanomechanics and sodium permeability of the endothelial surface layer modulated by hawthorn extract WS 1442. PLoS One.

[40] Le GA, Gavard J (2011). Role of endothelial cell-cell junctions in endothelial permeability. Methods Mol Biol.

[41] Komarova Y, Malik AB (2010). Regulation of endothelial permeability via paracellular and transcellular transport pathways. Annu Rev Physiol.

[42] Becker BF, Chappell D, Jacob M (2010). Endothelial glycocalyx and coronary vascular permeability: the fringe benefit. Basic Res Cardiol.

[43] Waanders F, de Vries LV, van GH, Hillebrands JL, Laverman GD, Bakker SJ (2011). Aldosterone, from (patho)physiology to treatment in cardiovascular and renal damage. Curr Vasc Pharmacol.

